# Percutaneous Coronary Intervention, Coronary Artery Bypass Surgery and the SYNTAX score: A systematic review and meta-analysis

**DOI:** 10.1038/srep43801

**Published:** 2017-03-02

**Authors:** Pravesh Kumar Bundhun, Chandra Mouli Yanamala, Feng Huang

**Affiliations:** 1 Institute of Cardiovascular Diseases, the First Affiliated Hospital of Guangxi Medical University, Nanning, Guangxi, 530021, P. R. China; 2 Department of Internal Medicine, EALING Hospital, University of Buckingham, Uxbridge road, Southall, UB1 3HW, London, United Kingdom

## Abstract

The SYNTAX [Synergy Between percutaneous coronary intervention (PCI) With Taxus and coronary artery bypass surgery (CABG)] score is a decision-making tool in interventional cardiology. However, several facts still remain to be addressed: What about PCI or CABG with a low versus a high score respectively? And what about PCI with a low score versus CABG with a high score? Electronic databases were carefully searched for relevant publications. Odds ratios (OR) with 95% confidence intervals (CIs) were calculated and the analysis was carried out by RevMan 5.3. Eleven studies with a total number of 11,037 patients were included. In terms of clinical outcomes, this analysis showed PCI to have significantly favored patients with a low versus a high SYNTAX score. In patients who were re-vascularized by CABG, mortality and major adverse cardiac events were significantly lower with a low SYNTAX score. However, when PCI with a low SYNTAX score was compared with CABG with a high SYNTAX score, no significant difference in mortality and combined death/stroke/myocardial infarction were observed. In conclusion, the SYNTAX score might be considered useful in interventional cardiology. Nevertheless, the fact that it has limitations when compared to newer tools should also not be ignored.

Cardiovascular disease (CVD), which might become the number one cause of death in the coming years, continues to affect a large population worldwide^1^. This chronic disease, which develops through different intravascular mechanisms in the elderly, and which often occurs as a long-term complication in patients with type 2 diabetes mellitus, is either treated by percutaneous coronary intervention (PCI) or coronary artery bypass surgery (CABG), based on several factors such as the severity of the disease, the health conditions and the preferences of the patients^2^,^3^.

New scientific research has shown SYNTAX [Synergy Between PCI With Taxus and CABG] score to be an essential decision-making tool in interventional cardiology^4^. In several recently published studies, PCI was suggested to be a more appropriate revascularization procedure in patients with a lower SYNTAX score whereas in patients with a high score, revascularization with CABG would probably be a better choice^5^. However, several facts still remain to be addressed: What about PCI or CABG with a low versus a high score respectively? And what about PCI with a low score versus CABG with a high score? In order to answer these interesting questions, we aimed to systematically carry out this meta-analysis.

## Methods

### Searched Databases and Searched Strategies

The following electronic databases: Medline (PubMed) database of medical research articles, EMBASE database and the Cochrane database of randomized controlled trials (RCTs) were carefully searched for English language publications [RCTs and observational studies (OS)] comparing:

- CABG versus PCI based on the SYNTAX score;
- PCI in patients with a low versus a high SYNTAX score;
- CABG in patients with a low versus a high SYNTAX score.

This search process was carried out using the terms ‘coronary artery bypass surgery, percutaneous coronary intervention, and the SYNTAX score’. Abbreviations of the above-mentioned terms: ‘CABG and PCI’ were also used during this search process. In addition, reference lists of relevant articles were also reviewed for suitable publications.

### Inclusion and Exclusion criteria

Studies were included if:

- They were randomized trials or observational research cohorts comparing CABG with PCI based on the SYNTAX score, or comparing PCI and CABG in patients with a low versus a high SYNTAX score respectively.
- They reported adverse clinical outcomes following the respective interventional or surgical procedures.

Studies were excluded if:

- They were meta-analyses, case studies or letters to editors, even though their main focus was on CABG, PCI and the SYNTAX score.
- They did not report adverse clinical outcomes following the corresponding interventional or surgical procedures.
- They were duplicates or they were associated with the same trials.
- They reported data (for example hazard ratios) in a form that could not be used in this current analysis.

### Definitions, reported outcomes and follow ups

The endpoints were:

- Mortality (all-cause death);
- Myocardial infarction (MI);
- Repeated revascularization including target vessel revascularization (TVR) and target lesion revascularization (TLR);
- Major adverse cardiac events (MACEs), defined as a combination of death, MI, and repeated revascularization. Since major adverse cerebrovascular and cardiovascular events (MACCEs) were reported only in one study, MACCEs and MACEs were combined together in one subgroup and analyzed;
- Death/stroke/MI;
- Stent thrombosis.

The respective outcomes and follow-up periods have been listed in Table 1.

### Data extraction and Review

(PKB and CMY) independently reviewed the trials and the OS which were qualified for this analysis. The authors’ names, the types of study reported (RCT or OS), the revascularization strategies (CABG or PCI) involved, the outcomes and the respective follow up periods reported, the total number of patients assigned to the PCI group, the CABG group, the low and the high SYNTAX score groups respectively, and data reporting the total number of events observed in the experimental and the control groups were carefully extracted by the same two authors. Any disagreement which followed was carefully discussed with the third author (FH) and a final decision was made. The bias risk observed among the trials (excluding OS) was assessed in accordance to the Cochrane Collaboration^6^, and the preferred reporting items in systematic reviews and meta-analyses (PRISMA) guideline was followed^7^.

### Statistical Analysis

Because studies are diverse clinically and methodologically in systematic reviews and meta-analyses, heterogeneity across the studies, should be expected. In general, heterogeneity assesses the null hypothesis to know if all the studies that have been included in an analysis are evaluating the same effect.

In this analysis, heterogeneity was assessed by two simple statistical methods: the Cochrane Q-statistic or chi-squared (χ^2^ or Chi^2^) test and the I^2^ statistic test.

Since this meta-analysis included only a small number of studies which might as a consequence, result in a low power of testing true heterogeneity, the I^2^ test was carried out in order to facilitate the assessment of inconsistency across the studies included.

I^2^ was calculated using this simple formula: I^2^ = 100% × [equation 1: (Q − df)/Q], whereby Q signified the Cochrane’s heterogeneity statistic and df signified the degree of freedom which could be represented by equation 2: (df = k − 1).

Negative I^2^ value if obtained (in exceptional cases), would automatically be changed to zero, so that the I^2^ value only remained between 0 and 100%.

An I^2^ value less than 25% represented a low heterogeneity, a value about 50% (greater than 25% but less than 75%) represented a moderate level of heterogeneity whereas an I^2^ value greater than 75% represented a high level of heterogeneity.

In addition, an I^2^ value equal to 50% was considered as a cut-off point for the use of a fixed effects model (I^2^ < 50%) or a random effects model (I^2^ > 50%) during the subgroup analysis.

It should also be noted that the interpretation of a given degree of heterogeneity across the studies which were analyzed might differ in accordance to whether the estimates showed a similar direction of effect.

In this analysis, a P value less than or equal to 0.05 (P ≤ 0.05) was considered significant statistically.

Odds ratios (OR) with 95% confidence intervals (CIs) were considered as the statistical parameters, and the pooled analysis was carried out with RevMan 5.3 software.

### Estimation of publication bias

This research consisted of only a small sample size of patients which were obtained from the few relevant studies which were selected for this analysis. Because of this reason, Begg’s and Egger’s tests as well as the Duval trim and fill methods were not effective to assess publication bias^8^. Instead, publication bias was estimated through funnel plots which were obtained from the Revman software.

## Results

### Outcome of the searched strategy

**Figure 1:**
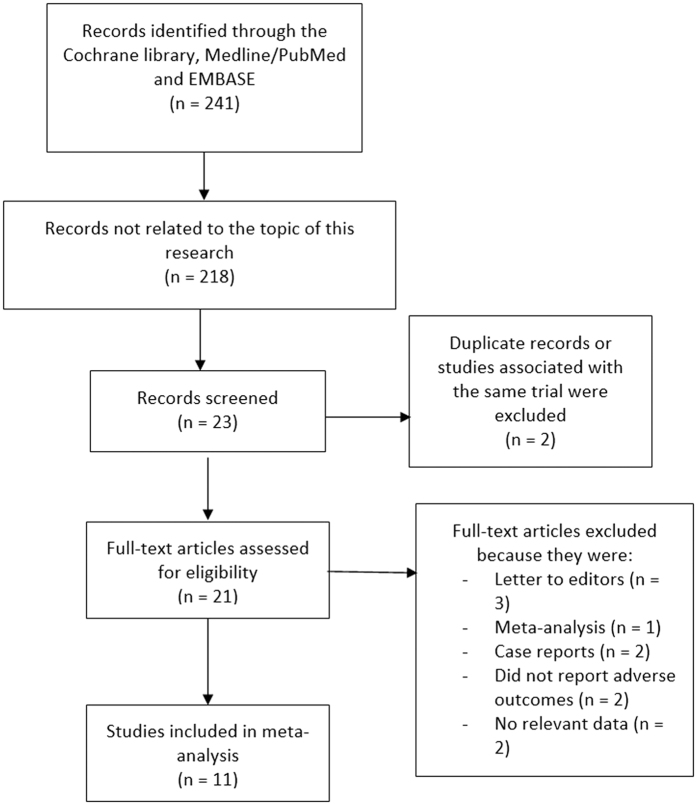
Flow diagram representing the study selection.

A total number of 241 publications were obtained from the electronic databases. Following a careful assessment of the titles and abstracts, 218 articles were eliminated since they were outside the scope of this research. In addition, another 10 articles were eliminated because they were: meta-analysis (1), letters to editors (3), case reports (2), studies that did not report the relevant clinical outcomes or data (4) associated with the same trial or cohort (2). Finally, only 11 relevant publications were included in this current analysis (Fig. 1).

### General features of the studies included

Eleven thousand and thirty-seven (11,037) patients [6893 patients assigned to the control group and 4144 patients assigned to the experimental group], which were obtained from 5 randomized trials and 6 OS were included in this analysis.

In order to answer the first question of this analysis, which was based on only those patients who were treated by PCI, 5521 patients with a low SYNTAX score were compared to 2531 patients with a high SYNTAX score;

To answer the second question of this analysis, which was based on those patients who were treated only by CABG, 1132 patients with a low SYNTAX score were compared with 773 patients with a high SYNTAX score;

To answer the third question of this analysis, 1200 patients with a low SYNTAX score who were assigned to PCI, were compared to 840 patients with a high SYNTAX score who were assigned to CABG.

When considering PCI with a low versus a high SYNTAX score, four studies [Garg^9^, Garg^10^, Wykrzykowska^11^ and Yadav^12^], which were classified to the same subgroup, involved a low score of ≤17, versus a high score above 17, whereas two studies [Capodanno^13^ and Kim^14^] which were included in another subgroup, involved a low score of ≤36 versus a high score above 34. Further details were summarized in Table 2.

### Baseline features of the studies included

The baseline features have been summarized in Table 3. The patients had a mean age which varied from 62.0 to 71.4 years (except study Wykrzykowska^11^ which included younger patients). All of the studies contained more male patients than female patients: (study Garg^9^: 72.9% versus 76.6%, study Garg^10^: 78.8% versus 74.1%, study Wykrzykowska^11^: 73.8% versus 73.8%, study Yadav^12^: 70.4% versus 66.0%, study Kim^14^: 51.4% versus 66.2%, study Cho^15^: 79.1% versus 73.6%, study Gannot^17^: 80.0% versus 82.0%, study Miyagi^18^: 85.7% versus 81.6%, study Capodanno^13^: 79.4% versus 75.9%, and study Shiomi^19^: 77.0% versus 71.0% male patients in the experimental versus the control group respectively). The percentage of patients with co-morbidities and other cardiovascular risk factors (hypertension, dyslipidemia, smoking, diabetes mellitus) was also listed (Table 3). The highest number of patients with hypertension (85% versus 86.0%) within both the experimental and control groups respectively, were observed in Study Shiomi^19^ whereas majority of patients with diabetes mellitus (53.0% versus 51.6%) within both the experimental and control groups respectively were observed in Study Miyagi^18^.

Nevertheless, even if the differences varied from study to study, there were almost no significant differences observed in the baseline features of patients who were assigned to the control (low score) and the experimental (high score) groups.

### Percutaneous coronary intervention in patients with a low versus a high SYNTAX score

**Figure 2:**
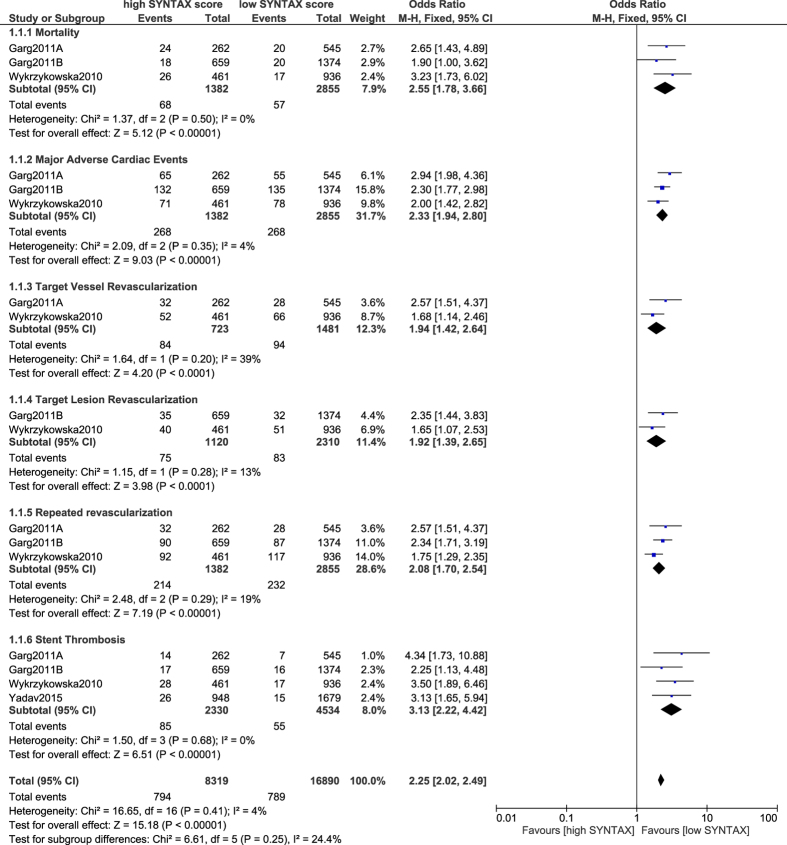
Outcomes associated with percutaneous coronary intervention following a low versus a high SYNTAX score.

At first, this analysis compared PCI in patients with a low versus a high SYNTAX score. In the beginning, a low score limit of ≤12, versus a high score limit of >12 was set. Results of this analysis showed PCI to have significantly favored patients with a low SYNTAX score whereby mortality, MACEs, TVR, TLR, repeated revascularization (combined TVR + TLR) and stent thrombosis were significantly lower (OR: 2.55, 95% CI: 1.78–3.66; P < 0.00001), (OR: 2.33, 95% CI: 1.94–2.80; P < 0.00001), (OR: 1.94, 95% CI: 1.42–2.64; P < 0.0001), (OR: 1.92, 95% CI: 1.39–2.65; P < 0.0001), (OR: 2.08, 95% CI: 1.70–2.54; P < 0.00001) and (OR: 3.13, 95% CI: 2.22–4.42; P < 0.00001) respectively as illustrated in Fig. 2.

**Figure 3:**
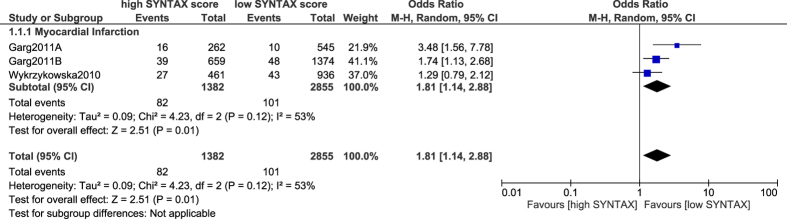
Myocardial infarction associated with percutaneous coronary intervention following a low versus a high SYNTAX score.

MI was also significantly higher with a high SYNTAX score (OR: 1.81, 95% CI: 1.14–2.88; P = 0.01) [Fig. 3].

**Figure 4:**
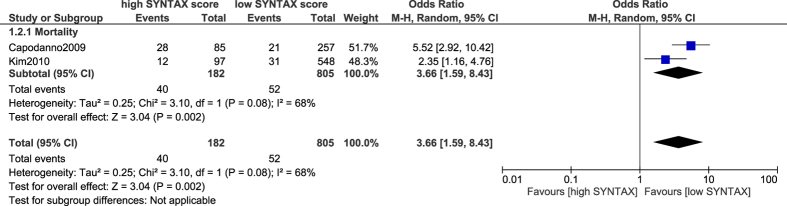
Mortality associated with percutaneous coronary intervention following a low versus a high SYNTAX score.

When a low score limit of ≤36 versus a high score limit of >34 was considered as the score range, mortality was still significantly higher in patients with a high SYNTAX score following PCI (OR: 3.66, 95% CI: 1.59–8.43; P = 0.002) as shown in Fig. 4.

### Coronary artery bypass surgery in patients with a low versus a high SYNTAX score

**Figure 5:**
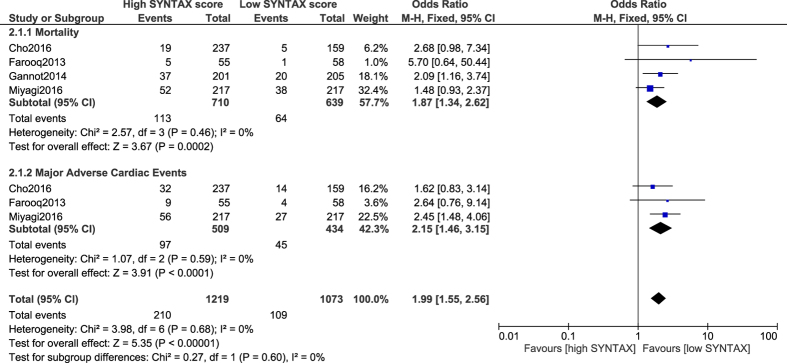
Outcomes associated with coronary artery bypass surgery following a low versus a high SYNTAX score.

Another analysis was carried out comparing CABG in patients with a low versus a high SYNTAX score. Results of this analysis showed that when a low score of <23, and a high score of ≥22 were considered, mortality and MACEs significantly favored a low SYNTAX score (OR: 1.87, 95% CI: 1.34–2.62; P = 0.0002) and (OR: 2.15, 95% CI: 1.46–3.15; P < 0.0001) respectively as shown in Fig. 5.

**Figure 6:**
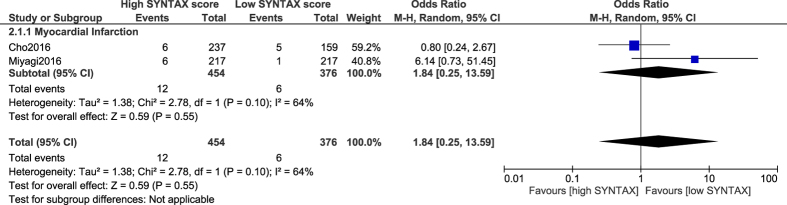
Myocardial infarction associated with coronary artery bypass surgery following a low versus a high SYNTAX score.

However, following CABG, the results showed MI not to be significantly different with a low versus a high SYNTAX score (OR: 1.84, 95% CI: 0.25–13.59; P = 0.55), but this specific subgroup was accompanied by a high level of heterogeneity (Fig. 6).

**Figure 7:**
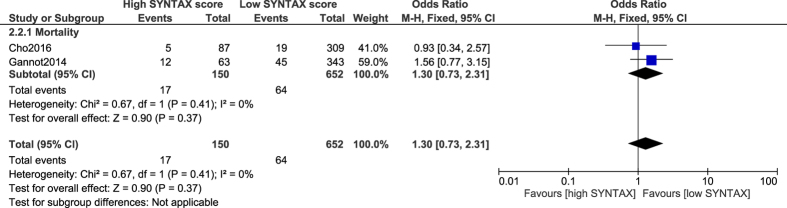
Mortality associated with coronary artery bypass surgery following a low versus a high SYNTAX score.

When a low score limit of <33, and a high score limit of ≥33 were considered, no significant difference was observed in mortality following CABG (OR: 1.30, 95% CI: 0.73–2.31; P = 0.37) [Fig. 7].

### Percutaneous coronary intervention with a low SYNTAX score versus coronary artery bypass surgery with a high SYNTAX score

Research has shown that the main purpose of the SYNTAX score was to stratify those patients who would benefit most from PCI and CABG respectively. According to literatures, PCI should be recommended to patients who were allocated a low score whereas CABG might be considered more appropriate to patients with a high score.

**Figure 8:**
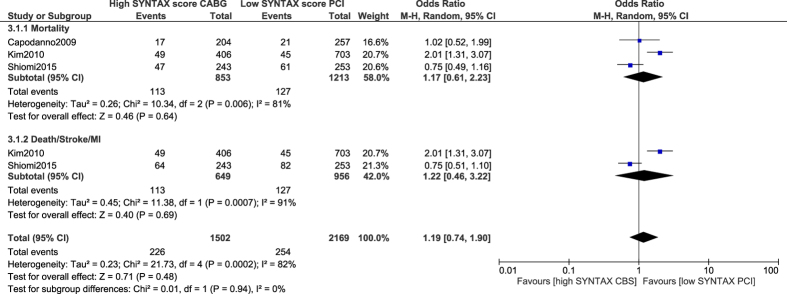
Outcomes associated with percutaneous coronary intervention in patients with a low SYNTAX score versus coronary artery bypass surgery in patients with a high SYNTAX score.

This current analysis has confirmed the statements published in previous literatures showing no significant difference in mortality, and the combined outcomes of death/stroke/MI observed between PCI with low SYNTAX score versus CABG with high SYNTAX score, (OR: 1.17, 95% CI: 0.61–2.23; P = 0.64), and (OR: 1.22, 95% CI: 0.46–3.22; P = 0.69) respectively as shown in Fig. 8.

**Figure 9:**
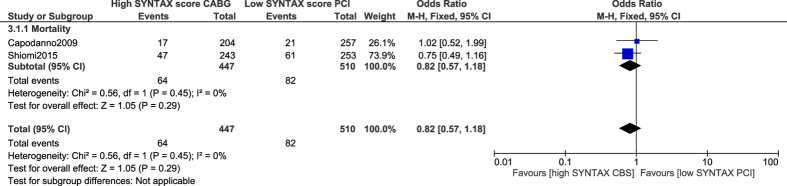
Mortality associated with percutaneous coronary intervention in patients with a low SYNTAX score versus coronary artery bypass surgery in patients with a high SYNTAX score.

Study Kim^14^ was thought to have possibly been introducing bias within this current analysis of PCI versus CABG. Therefore, another analysis was carried out but this time without including study Kim^14^. Nevertheless, a similar mortality rate observed between PCI with a low SYNTAX score versus CABG with a high SYNTAX score (OR: 0.82, 95% CI: 0.57–1.18; P = 0.29) [Fig. 9] and with a very low level of heterogeneity further confirmed the above-shown result.

**Figure 10:**
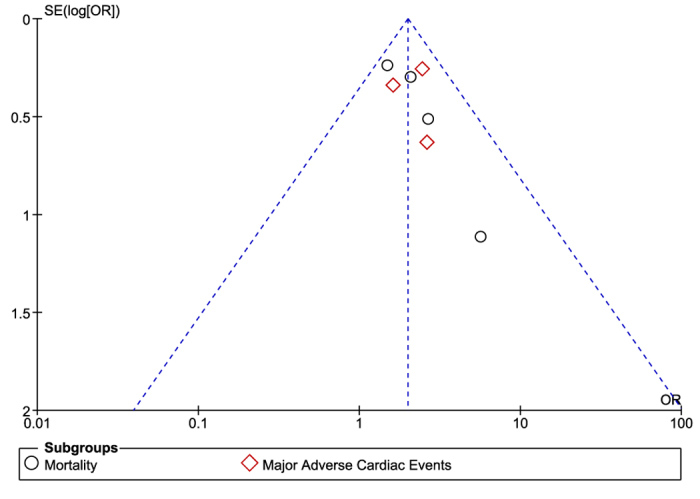
Funnel plot showing publication bias.

**Figure 11:**
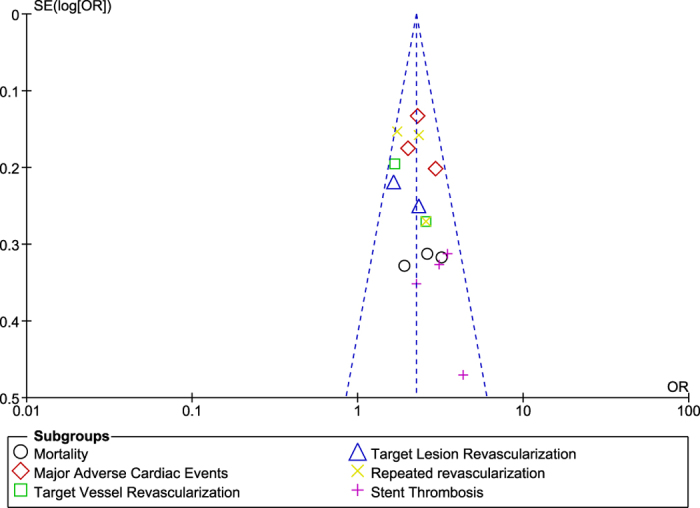
Funnel plot showing publication bias.

Based on a visual inspection of the funnel plots obtained through RevMan, a low level of publication bias was observed among most of the studies which were involved while carrying out this analysis (Figs 10 and 11).

## Discussion

This study aimed to answer three major questions: the outcomes following PCI with a low versus a high SYNTAX score, the outcomes following CABG with a low versus a high SYNTAX score, and the outcomes following PCI with a low SYNTAX score versus CABG with a high SYNTAX score.

Results of this analysis showed a low SYNTAX score to be associated with significantly lower adverse outcomes compared to a high SYNTAX score following PCI (20% versus 49.2% for mortality, 9.4% versus 19.4% for MACEs, 6.3% versus 11.6% for TVR, 3.6% versus 6.7% for TLR, 3.53% versus 5.93% for MI, and 1.21% versus 3.65% for stent thrombosis), clearly answering the first question.

Following revascularization with CABG, a low SYNTAX score was associated with significantly lower mortality (10.0% versus 15.9%) and MACEs (10.4% versus 19.1%) when compared to a high score, providing an answer to the first part of the second question. However, when a higher limit of scores was considered, no significant difference was observed in the mortality rate (9.82% versus 11.3%), further answering the second question. Comparing a low score with a high score showed the former to be associated with a favorable outcome. However, when two high score limits were compared (for example, a low score of <34 was compared with a high score of >34), the difference was not significant.

In response to the third question, the current results showed that when PCI with a low SYNTAX score was compared to CABG with a high SYNTAX score, a similar mortality rate (16.1% versus 14.3%) was observed following the respective revascularization procedures.

To further support this analysis, the SHINANO registry showed SYNTAX score to be beneficial in predicting the incidence of MACEs following PCI^20^. That particular registry included patients with prior heart failure and coronary artery disease. A high SYNTAX score was associated with higher prediction of MACEs. According to what they have observed in their results, the authors suggested SYNTAX score to be a useful parameter in order to improve risk stratification in patients with complex coronary diseases.

In addition, the five-year follow-up of the SYNTAX trial showed that in patients who were allocated a low SYNTAX score, PCI was an acceptable revascularization strategy when compared to CABG; but with a high rate of late repeated revascularization. Other studies showed patients with low SYNTAX scores to have similar rates of MACCEs following either PCI or CABG further supporting this current analysis^21^.

Our results showed PCI with a low SYNTAX score to be comparable to CABG with a high SYNTAX score. To again support this point, the Evaluation of XIENCE versus Coronary Artery Bypass Surgery for Effectiveness of Left Main Revascularization (EXCEL) trial which randomly assigned 1905 patients with left main coronary disease showed that PCI with everolimus eluting stents was non-inferior to CABG^22^.

Nevertheless, even if a recently published systematic review showed that the SYNTAX score was comparable to the clinical SYNTAX scoring system in predicting soft endpoints such as TVR and MACEs, the same study stated that clinical SYNTAX score was even better in predicting prognosis since it involved patients with important clinical risk factors^23^. Another study showed the Logistic Clinical SYNTAX score to further enhance the prediction of mortality following PCI^24^.

The SYNTAX score II which is another potential tool combining clinical predictors with anatomical factors showed robust and more accurate prognosis compared to the SYNTAX score^25^. The benefits of the SYNTAX score II were further supported by other studies especially in patients with left main coronary diseases and other complex coronary artery diseases^26^,^27^.

Furthermore, the New Risk Stratification (NERS) score which encompasses clinical, procedural and angiographic characteristics showed a higher predictive ability of MACEs when compared to the SYNTAX score^28^. However, new research should be expected to boost/support and further address these comments^29^.

## Conclusion

Since a high SYNTAX score was associated with significantly higher adverse outcomes irrespective of which revascularization procedure was involved (PCI or CABG), and because no significant difference in mortality was observed with a low SYNTAX score following PCI compared to a high SYNTAX score following CABG, the SYNTAX score should be considered among the important decision-making tools which might be used to stratify patients who would most probably benefit from PCI and CABG respectively, or to select patients who would probably be susceptible to unfavorable clinical outcomes following these revascularization procedures. Nevertheless, we should also not ignore the fact that the SYNTAX score has limitations when compared to newer tools.

## Additional Information

**How to cite this article**: Bundhun, P. K. *et al*. Percutaneous Coronary Intervention, Coronary Artery Bypass Surgery and the SYNTAX score: A systematic review and meta-analysis. *Sci. Rep.* **7**, 43801; doi: 10.1038/srep43801 (2017).

**Publisher's note:** Springer Nature remains neutral with regard to jurisdictional claims in published maps and institutional affiliations.

## Figures and Tables

**Table 1:** Reported adverse outcomes and follow-up periods.

Studies	Outcomes reported	Follow-up periods
Garg^9^	Death, MI, MACEs, TVR, definite and probable ST	12 months
Garg^10^	Death, MI, revascularization, TLR, MACEs, definite or probable ST	12 months
Wykrzykowska^11^	Death, MI, TVR, TLR, ST, MACEs	12 months
Yadav^12^	Definite and probable ST	30 days, 12 months
Kim^14^	Death, MI, stroke	3 years
Capodanno^13^	Death	2 years
Cho^15^	Death, stroke, MI, revascularization, MACCEs	10 years
Farooq^16^	Death, stroke, MI, revascularization	4 years
Gannot^17^	Death	8 years
Miyagi^18^	Death, revascularization, MACEs, MI	5 years
Shiomi^19^	Death, MI, stroke, revascularization	5 years

Abbreviations: MI: myocardial infarction, MACEs: major adverse cardiac events, TVR: target vessel revascularization, TLR: target lesion revascularization, ST: stent thrombosis, MACCEs: major adverse cerebrovascular and cardiovascular events.

**Table 2:** General features of the studies included.

Study	Type of study	Low syntax range	High syntax range	No of patients with low score (n)	No of patients with high score (n)
PCI with low versus high SYNTAX score					
Garg^9^	RCT	≤16	>16	545	262
Garg^10^	RCT	≤17	>17	1374	659
Wykrzykowska^11^	RCT	≤16	>16	936	461
Yadav^12^	RCT	≤12	>12	1679	948
Capodanno^13^	OS	≤34	>34	257	85
Kim^14^	OS	≤36	>36	703	116
Total no of patients (n)				**5521**	**2531**
CABG with low versus high SYNTAX score					
Cho^15^	OS	<33	≥33	309	87
Cho^15^	OS	<23	≥23	159	237
Farooq^16^	RCT	<22	≥22	58	55
Gannot^17^	OS	<33	≥33	343	63
Gannot^17^	OS	<23	≥23	205	201
Miyagi^18^	OS	≤23	>23	217	217
Total no of patients (n)				**1132**	**773**
Low score PCI versus high score CABG					
Capodanno^13^	OS	≤34	>34	257	204
Kim^14^	OS	≤36	>36	703	406
Shiomi^19^	OS	<33	≥33	240	230
Total no of patients (n)				**1200**	**840**

Abbreviations: RCT: randomized controlled trials, OS: observational studies, PCI: percutaneous coronary intervention, CABG: coronary artery bypass surgery, SYNTAX: Synergy Between PCI With Taxus and Cardiac Surgery.

**Table 3:** Baseline features of the patients involved.

Studies*	Age (years)	Males (%)	HT (%)	Ds (%)	Cs (%)	DM (%)
	Exp/ctrl	Exp/ctrl	Exp/ctrl	Exp/ctrl	Exp/ctrl	Exp/ctrl
Garg^9^	66.1/62.6	72.9/76.7	59.8/55.0	39.5/39.8	32.4/40.8	18.7/11.4
Garg^10^	65.5/63.0	78.8/74.1	68.3/71.0	59.8/64.9	29.1/27.4	24.7/21.3
Wykrzykowska^11^	51.8/46.4	73.8/73.8	70.3/75.5	61.8/67.1	27.3/27.3	24.1/22.4
Yadav^12^	62.6/59.7	70.4/66.0	66.0/63.4	55.8/56.4	31.1/37.7	30.3/27.5
Kim^14^	66.9/58.5	51.4/66.2	59.5/42.5	36.1/28.7	18.5/26.9	40.8/22.1
Cho^15^	67.4/66.8	79.1/73.6	—	34.9/47.8	20.9/18.4	51.2/48.1
Gannot^17^	69.0/65.0	80.0/82.0	65.0/64.0	54.0/61.5	6.00/6.50	35.0/35.0
Miyagi^18^	71.0/71.0	85.7/81.6	84.8/79.7	80.2/79.3	22.1/17.5	53.0/51.6
Capodanno^13^	68.1/66.1	79.4/75.9	79.4/68.1	53.4/56.4	51.0/45.1	50.0/30.7
Shiomi^19^	69.4/71.4	77.0/71.0	85.0/86.0	—	25.0/21.0	45.0/42.0

Abbreviations: Exp: experimental group/high SYNTAX score, Ctrl: control group/low SYNTAX score, HT: hypertension, Ds: dyslipidemia, Cs: current smoker, DM: diabetes mellitus.

^*^No baseline feature was provided for study Farooq^16^.
